# Development and validation of a nomogram model for the prediction of recurrence after endoscopic sinus surgery in patients with chronic rhinosinusitis with nasal polyps

**DOI:** 10.3389/fsurg.2025.1581417

**Published:** 2025-04-25

**Authors:** Ruiying Ma, Zhibin Lu, Jianguang Zhu, Yundong Bai, Xuyan Wang

**Affiliations:** Department of ENT (ear-nose-throat), The 82nd Group Army Hospital of the Chinese People’s Liberation Army, Baoding, Hebei, China

**Keywords:** endoscopic sinus surgery, recurrence, chronic rhinosinusitis with nasal polyps, nomogram model, risk factors

## Abstract

**Objective:**

To analyze the factors influencing recurrence after endoscopic sinus surgery (ESS) in patients with chronic rhinosinusitis with nasal polyps (CRSwNP). A nomogram model for risk prediction was constructed and validated.

**Methods:**

Retrospective analysis of clinical data of 460 patients with chronic sinusitis and nasal polyps at the 82nd Group Military Hospital of the People’s Liberation Army of China from January 2020 to May 2022. Randomly divide into training set (*n* = 322) and validation set (*n* = 138) in a 7:3 ratio. Divide the training set into a Recurrence group or a Non-recurrence group based on whether the patient relapses within one year after surgery. Analysis the risk factors of postoperative recurrence in patients and draw a risk prediction nomogram model.

**Results:**

Gastroesophageal reflux disease (GERD), bronchial asthma, sinusitis type, allergic rhinitis, eosinophil count, neutrophil count, lymphocyte count, and total sinus score were independent risk factors for postoperative recurrence in CRSwNP patients (*P* < 0.05). The Nomogram model constructed based on the above factors was validated, and the results showed that the C-indices of the training set and validation set are 0.935 and 0.923, respectively. The internal validation receiver operating characteristic (ROC) curve of area under the curve (AUC) was 0.948; The external validation set AUC is 0.932. The decision curve shows a higher net benefit value when the threshold probability is between 5% and 100%.

**Conclusions:**

The predictive nomogram model constructed in this article has high recognition efficiency.

## Introduction

Chronic rhinosinusitis with nasal polyps (CRSwNP) is a common chronic inflammatory disease of the nasal cavity or nasal mucosa, with a long course and susceptibility to multiple occurrences (1). Epidemiological studies in China have shown that the incidence rate of CRSwNP is between 8% and 15%, and it has been increasing annually in recent years (1, 2). Nasal congestion, headache, and olfactory dysfunction are the main clinical symptoms (2, 3). Failure to diagnose and treat in a timely manner can increase the deterioration of respiratory infections, reduce the quality of life of patients, and, in severe cases, lead to brain abscesses, purulent meningitis, and even death (1–3). At present, ESS is commonly performed to remove lesions, maximally preserve normal mucosa, restore nasal function, and improve nasal ventilation (4, 5). However, the recurrence rate after surgery is still as high as 40%–60%, which not only reduces the patient’s willingness to undergo treatment but also increases the risk of needing a second treatment and the economic burden (5–7). Therefore, we plan to explore the risk factors for recurrence after ESS in CRSwNP patients and to construct a prognostic prediction model to discover new high-risk populations and provide new ideas for clinical treatment.

## Materials and Methods

### General information

A retrospective analysis was performed to select CRSwNP patients who underwent ESS at the 82nd Group Army Hospital of the People’s Liberation Army between January 2020 and May 2022.

### Inclusion criteria

- Meets the diagnostic criteria for CRSwNP, and the imaging CT image shows a sinus meatus complex with or without inflammatory lesions of the sinus mucosa;
- Age ≥18 years and less than 70 years;
- Complete clinical data;
- Patients who have undergone their first successful ESS.

### Exclusion criteria

- Severe heart, lung, liver, and kidney dysfunction, as well as immune-related diseases;
- Patients with concomitant malignant tumours and psychiatric disorders;
- Women who are breastfeeding or pregnant;
- Patients who did not cooperate with the follow-up examination.

### Data collection

Researchers used the hospital’s electronic health record system to collect general information about patients. The data included the following: (1) General information, such as sex, age, body mass index (BMI), smoking history and drinking history. (2) Underlying diseases, such as hypertension, diabetes, gastroesophageal reflux, sinusitis, bronchial asthma, coronary heart disease, and allergic rhinitis, were recorded. (3) Blood indicators, such as eosinophil count (×10^9^/L), neutrophil count (×10^9^/L), lymphocyte count (×10^9^/L), haemoglobin (g/L), and platelet count (×10^9^/L), were measured. Abdominal blood was collected from the patient on the first day before surgery, and the sample was placed in a vacuum anticoagulant tube and allowed to rest at room temperature for 15 min. The sample was centrifuged at 3000 r/min with a centrifugal radius of 12 cm. After 10 min, the serum was collected and separated as the test sample. A BK-200 model fully automatic biochemical analyser (Shandong Boke Scientific Instrument Co., Ltd.) was used to detect the above indicators. (4) Preoperative condition evaluation: The visual analogue VAS score was used to assess the degree of pain in patients, with a maximum score of 10 points. The higher the score is, the greater the degree of pain in the patient. The CT scan results of the sinuses were combined with the Lund Mackay scoring criteria to score the maxillary sinus (M), anterior ethmoid sinus (AE), posterior ethmoid sinus (PE), sinus meatus complex (OMC), and total score. Evaluations were based on the following scoring criteria: complete sinus turbidity, partial turbidity, and no turbidity corresponded to 2, 1, and 0 points, respectively; the OMC blockage score was 2 points; the partial blockage score was 1 point; and the unobstructed score was 0 points. The sum of all the scores is the total score.

### Surgical method

All patients underwent CT and sinus endoscopy examinations. The patient’s blood pressure and blood sugar were monitored one week before surgery, and oral antibiotics were administered. First, adrenaline was used to contract the nasal mucosa, the nasal cavity was thoroughly opened, and the nasal polyp was removed. The second step involves removing the nasal polyp, exposing the outer wall of the nose, opening the mucosa, and exposing the sinuses, anterior sinuses, and nasal cavity. The third step involves opening the sphenoid sinus and posterior ethmoid sinus appropriately under ESS, on the basis of CT images and specific conditions during ESS, to remove the lesion. After surgery, the wound was cleaned with a large amount of physiological saline and filled with Vaseline gauze strips. Twelve to twenty-four hours after surgery, the Vaseline gauze strip was removed. On the 4th day after surgery, blood clots and secretions were removed from the nasal cavity, and the oral cavity was cleared. Postoperative examination of the patient’s condition revealed the removal of residual polyps, particles, and blisters and improved nasal sinus mucosal epithelialization.

### Recurrence assessment criteria

The patient complained of nasal foreign body sensation and symptoms such as runny nose and nasal congestion during the postoperative follow-up exam. CT examination revealed soft tissue shadows in the nasal cavity, high density in the sinuses, and increased mucosa.

### Grouping method

The included patients will be randomly divided into a training set and a validation set in a 7:3 ratio. According to the recurrence assessment criteria, patients are divided into a Recurrence group and a Non-recurrence group. Analyze independent risk factors for recurrence after ESS in CRSwNP patients using patient data from the training set and construct a predictive nomogram model. Perform external validation on the nomogram model using validation set data.

### Statistical methods

Data were input into Microsoft Excel and analysed via SPSS version 26.0 (IBM Corp, Armonk, NY, USA). Data that do not follow a normal distribution were expressed as median and the interquartile range (IQR) and analyzed with Mann–Whitney *U* test. Count data were expressed as percentages, and chi-square tests were performed between groups. Through univariate and multivariate logistic regression analyses, independent risk factors for recurrence after ESS in CRSwNP patients were identified. After statistical processing, *P* < 0.05 was considered statistically significant. A nomogram model was constructed via the R (R 3.5.3) software package and the RMS package, the consistency index (C-index) was calculated by using the RMS package, and calibration curves and receiver operating characteristic (ROC) curves were drawn to evaluate the predictive performance of the model.

## Results

**Figure 1:**
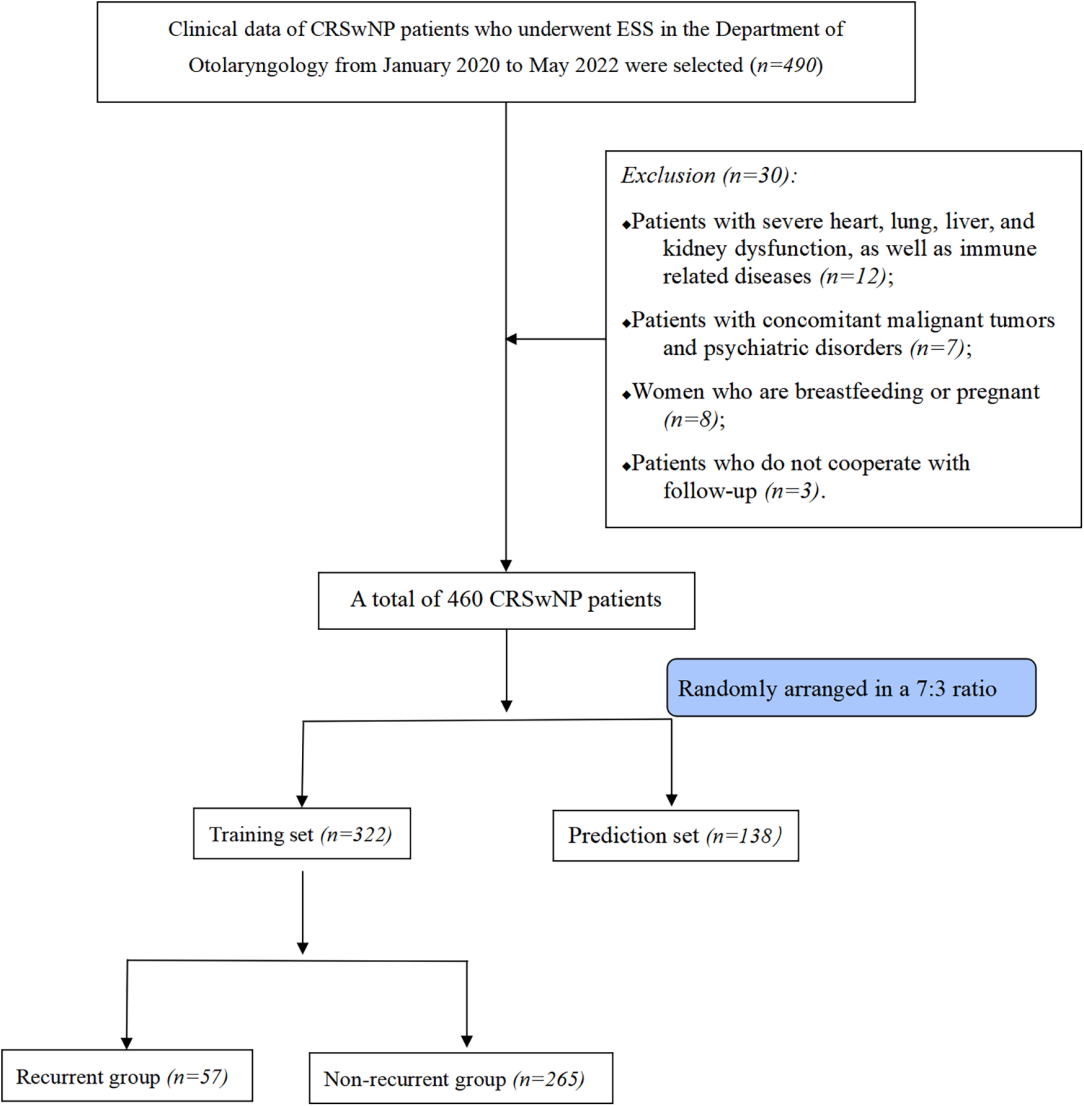
Flowchart of the selection process for this research subject; CRSwNP, chronic rhinosinusitis with nasal polyps; ESS, endoscopic sinus surgery.

This study included a total of 322 patients with CRSwNP who underwent ESS, including 205 males and 117 females. Their ages ranged from 24 to 72 years, with a median of 49 [42, 55] years. Among them, 57 patients experienced postoperative recurrence, with an incidence rate of 17.7% (57/322). The flowchart of the selection process for the participants is shown in Figure 1 and participant characteristics is shown in Table 1.

**Table 1:** Participant characteristics.

Characteristics	Value (*n* = 322)
Gender, Male	205 (63.7)
Age (years)	49 [42, 55]
BMI (kg/m^2^)	23.2 [21.4, 55.0]
Smoking history (yes)	111 (34.5)
Drinking history (yes)	104 (32.3)
Diabetes history (yes)	48 (14.9)
GERD (yes)	100 (31.1)
bronchial asthma (yes)	50 (15.5)
coronary heart disease (yes)	73 (22.7)
Disease course (year)	5 [3, 6]
Sinusitis type	
Ⅰ	174 (54.0)
Ⅱ	109 (33.9)
Ⅲ	39 (12.1)
Allergic rhinitis (yes)	71 (22.0)
Eosinophils (×10^9^/L)	0.95 [0.68, 1.21]
Neutrophils (×10^9^/L)	47.00 [38.75, 53.00]
Lymphocyte (×10^9^/L)	1.20 [0.90, 1.50]
Haemoglobin (g/L)	104 [93,124]
Platelet (×10^9^/L)	284.00 [253.25, 298.25]
Preoperative VAS score (points)	5 [4, 6]
Total CT score of sinuses (points)	13 [11, 16]

Note: Data were presented as *n* (%) or median [IQR]. CRSwNP, chronic rhinosinusitis with nasal polyps; ESS, endoscopic sinus surgery; BMI, body mass index; GERD, gastroesophageal reflux disease.

The recurrent group and the nonrecurrent group were significantly different in terms of age; GERD; diabetes; bronchial asthma; sinusitis classification; allergic rhinitis; eosinophil, neutrophil, lymphocyte counts; preoperative visual analogue scale (VAS) score; and total sinus score (*P* < 0.05) (Table 2).

**Table 2:** Univariate analysis of postoperative recurrence after ESS in CRSwNP patients.

Factor	Recurrent group (*n* = 57)	Non recurrent group (*n* = 265)	*χ* ^2^ */Z*	*P*
Male (yes)	31 (54.39)	174 (65.66)	2.578	0.108
Age (years)	52 [45.5, 59.5]	48 [42, 54]	−267.3	0.008
BMI (kg/m^2^)	23.40 [21.50, 25.75]	22.8 [21.4, 24.5]	−1.551	0.121
Smoke (yes)	25 (43.86)	86 (32.45)	2.702	0.1
Drink (yes)	20 (35.09)	84 (31.70)	0.246	0.62
GERD (yes)	33 (57.89)	67 (25.28)	23.301	<0.001
Diabetes (yes)	18 (31.58)	30 (11.32)	15.177	<0.001
bronchial asthma (yes)	23 (40.35)	27 (10.19)	32.536	<0.001
coronary heart disease (yes)	17 (29.82)	56 (21.13)	2.022	0.155
Disease course (year)	5 [3, 6]	5 [3, 6]	−0.905	0.366
Sinusitis type			45.006	<0.001
Ⅰ	15 (26.32)	159 (60.00)		
Ⅱ	21 (36.84)	88 (33.21)		
Ⅲ	21 (36.84)	18 (6.79)		
Allergic rhinitis (yes)	29 (50.88)	42 (15.85)	60.99	<0.001
Eosinophils (×10^9^/L)	1.04 [0.85, 1.25]	0.92 [0.65, 1.15]	−3.365	0.001
Neutrophils (×10^9^/L)	53.0 [46.5, 62.0]	47 [37, 52]	−5.628	<0.001
Lymphocyte (×10^9^/L)	1.1 [0.8, 1.2]	1.2 [1.0, 1.5]	−4.219	<0.001
Haemoglobin (g/L)	105.0 [94.5, 124.5]	104 [92, 124]	−0.987	0.324
Platelet (×10^9^/L)	284 [258, 297]	274 [247, 299]	−0.528	0.598
Preoperative VAS score (points)	5.0 [4.5, 6.0]	5 [4, 6]	−2.330	0.020
Total CT score of sinuses (points)	15 [13, 18]	13 [11, 15]	−4.481	<0.001

Note: Data were presented as n (%) or median [IQR]. CRSwNP, chronic rhinosinusitis with nasal polyps; ESS, endoscopic sinus surgery; BMI, body mass index; GERD, gastroesophageal reflux disease.

The dependent variable was whether postoperative recurrence occurred, and the independent variable was the factor that was statistically significant for postoperative recurrence according to the univariate analysis. Logistic multiple stepwise regression analysis was performed after assignment. The results revealed that GERD, bronchial asthma, sinusitis classification, allergic rhinitis, eosinophil count, neutrophil count, lymphocyte count, and total sinus score were independent risk factors for recurrence after ESS in CRSwNP patients (*P* < 0.05) (Table 3).

**Table 3:** Multivariate logistic regression analysis of recurrence after ESS in CRSwNP patients.

Factor	*B*	*SE*	Wald *χ*^2^	*P*	*OR*	95% CI
GERD	1.975	0.518	14.561	<0.001	7.21	2.614–19.889
Bronchial asthma	1.362	0.563	5.848	0.016	3.903	1.294–11.767
Sinusitis type			18.198	<0.001		—
Sinusitis type II	1.137	0.577	3.886	0.049	3.119	1.007–9.663
Sinusitis type III	2.808	0.658	18.198	<0.001	16.57	4.561–60.195
Allergic rhinitis	2.206	0.543	16.522	<0.001	9.076	3.133–26.290
Eosinophils count	2.632	0.933	7.952	0.005	13.9	2.231–86.589
Neutrophils count	0.158	0.032	23.953	<0.001	1.171	1.099–1.247
Lymphocyte count	−1.847	0.774	5.687	0.017	0.158	0.035–0.720
Total score of sinuses	0.195	0.071	7.478	0.006	1.215	1.057–1.397
Constant	−17.568	3.25	29.214	<0.001	0.000	—

Note: CRSwNP, chronic rhinosinusitis with nasal polyps; ESS, endoscopic sinus surgery; GERD, gastroesophageal reflux disease.

**Figure 2:**
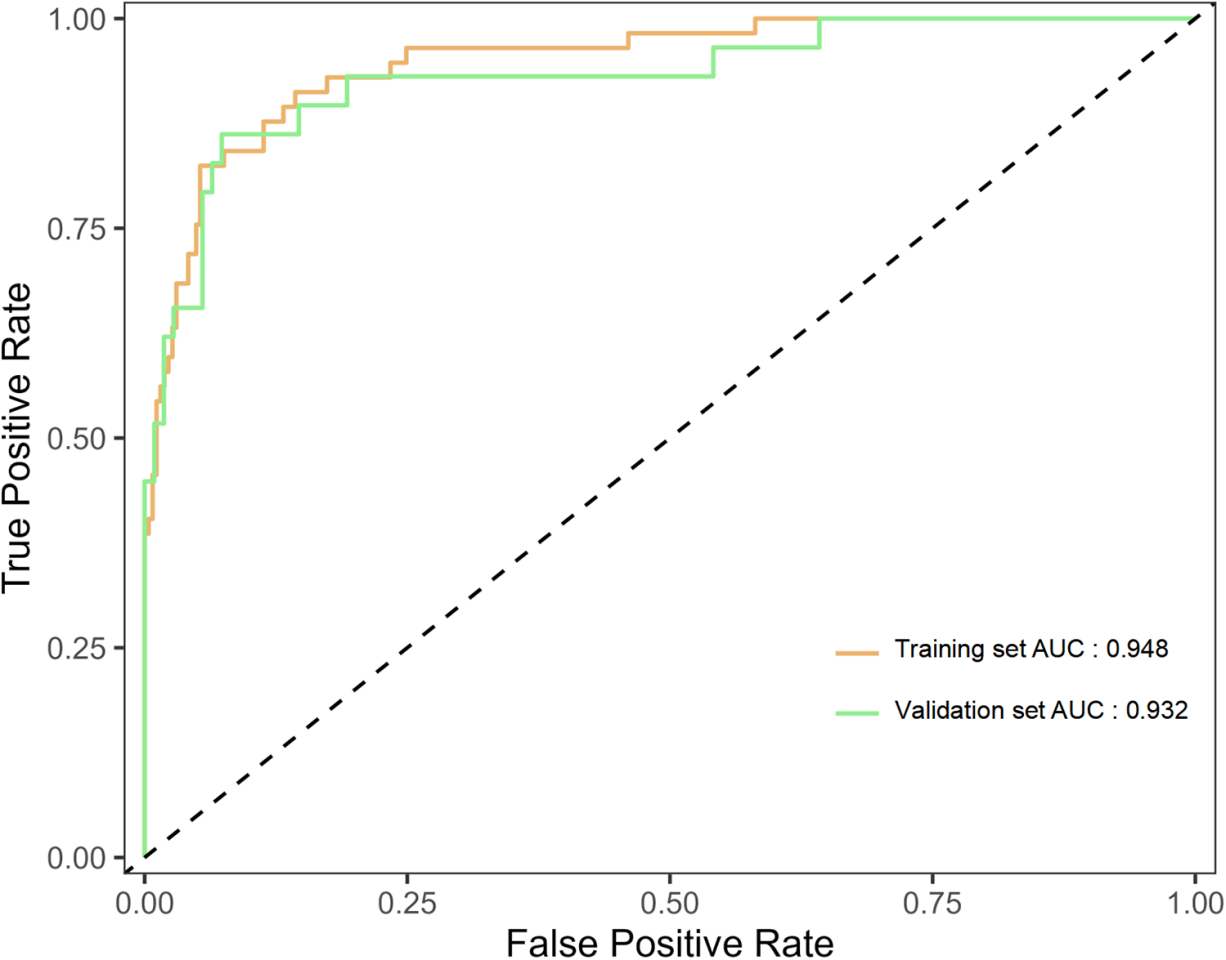
Nomogram model for predicting postoperative recurrence in CRSwNP patients; CRSwNP, chronic rhinosinusitis with nasal polyps; GERD, gastroesophageal reflux disease.

**Figure 3:**
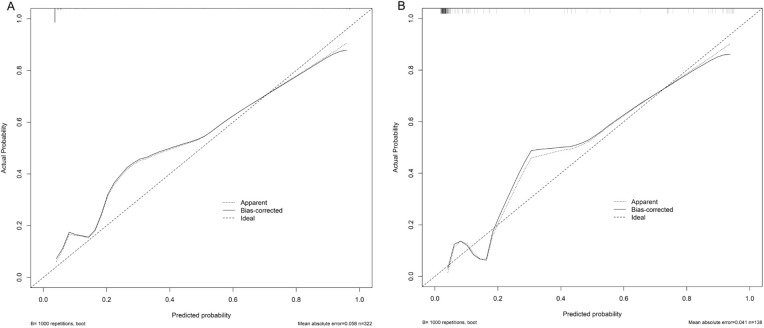
Validation of the nomogram model for predicting postoperative recurrence in CRSwNP patients; CRSwNP, chronic rhinosinusitis with nasal polyps. **(A)** Is the training set; **(B)** Is the validation set.

**Figure 4:**
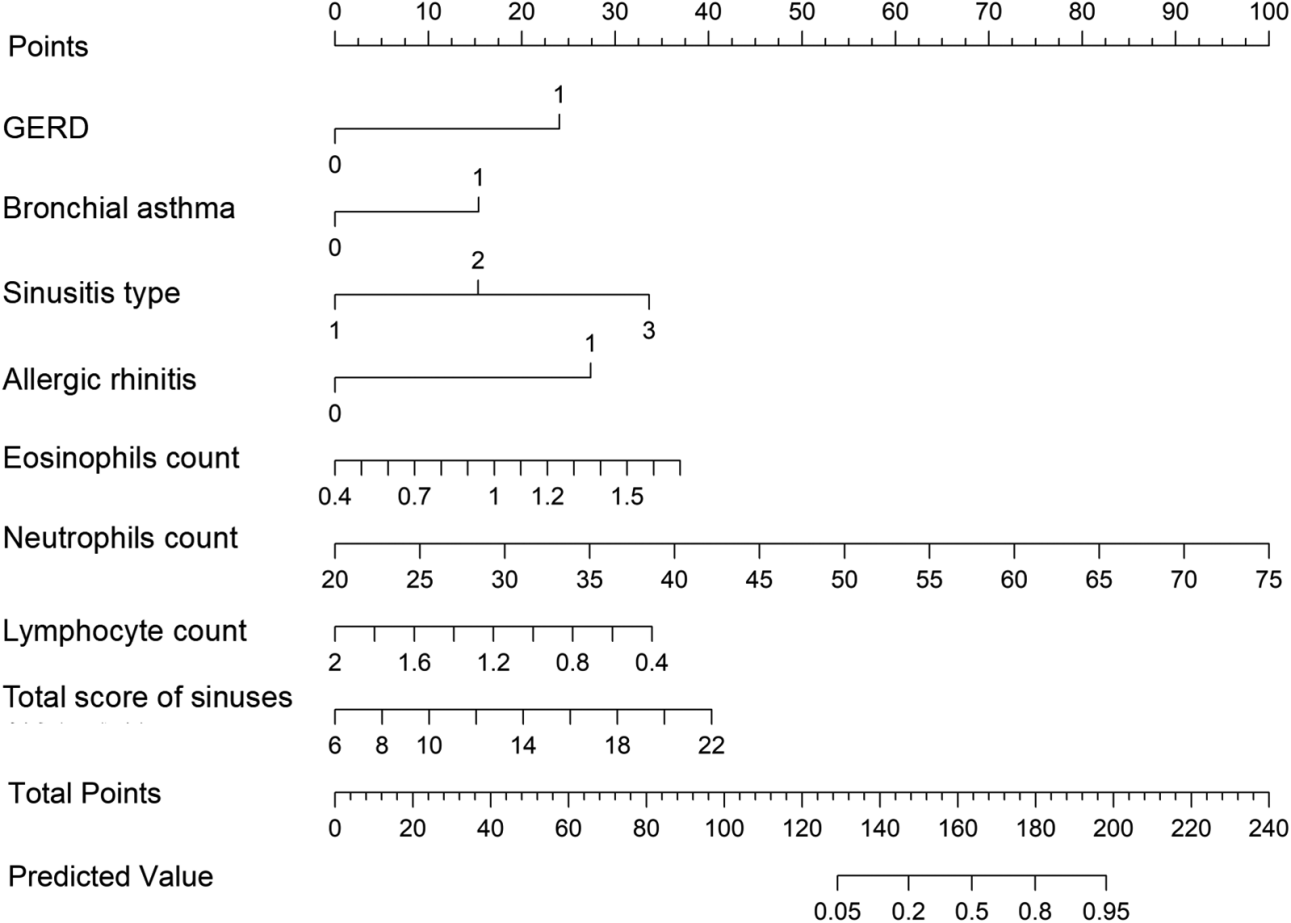
ROC curve of the nomogram model for predicting postoperative recurrence in CRSwNP patients; ROC, receiver operating characteristic; CRSwNP, chronic rhinosinusitis with nasal polyps.

**Figure 5:**
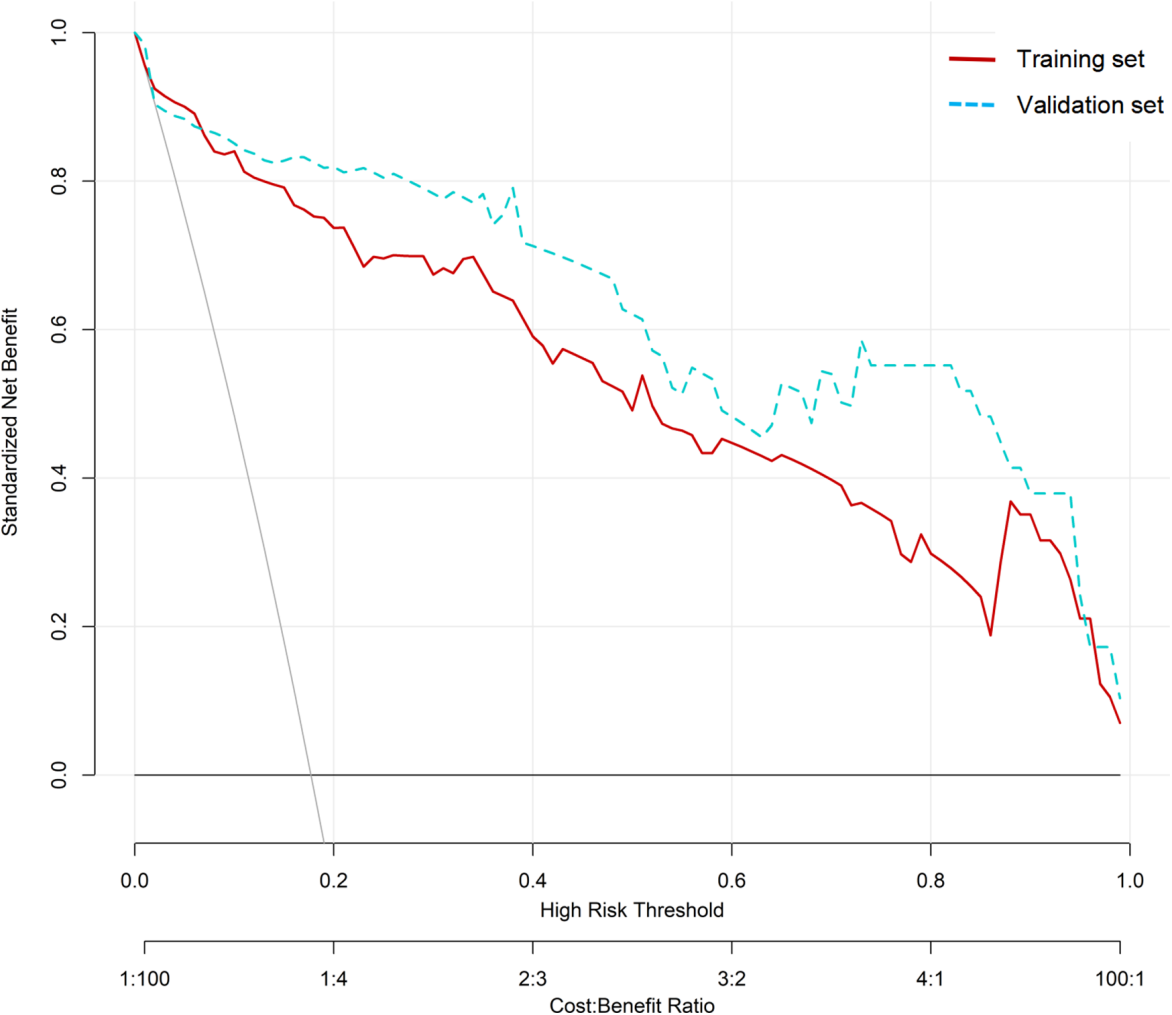
Decision curve of nomogram model.

A nomogram model for predicting recurrence risk after ESS in CRSwNP patients was established on the basis of 8 independent risk factors, as shown in Figure 2. Internal validation of the nomogram model was performed using Bootstrap method (after 1000 repeated samplings of raw data), while external validation was performed using a validation set. The results showed that the C-indexs of the training set and validation set were 0.935 (95% CI: 0.902–0.968) and 0.923 (95% CI: 0.865–0.980), respectively. The calibration curves of the two sets were close to the diagonal (ideal curve), as shown in Figure 3. The area under the ROC curve (AUC) for internal validation was 0.948 (95% CI: 0.918–0.978), with a sensitivity of 68.4% and a specificity of 95.8%; The external validation AUC was 0.932 (95% CI: 0.873–0.990), with a sensitivity of 79.3% and a specificity of 93.6% (Figure 4). The *P*-values of the Hosmer Lemeshow goodness of fit test for the modeling group and validation group were 0.844 and 0.086, respectively, indicating good model calibration. The decision curve shows that there is a higher net benefit value when the threshold probability is between 5% and 100% (Figure 5).

## Discussion

The results of this study revealed that the incidence of recurrence after ESS in CRSwNP patients was 17.7%. Moreover, Yang et al. (8) reported that the recurrence rate of CRSwNP after ESS was 18.1%. Xu Kairui’s (9) research also revealed that the recurrence rate of CRSwNP after ESS is as high as 44.2%. Reducing the incidence of recurrence after ESS for CRSwNP is still challenging. This study analysed the clinical data of 322 patients with CRSwNP who underwent ESS. After logistic regression analysis, the results revealed that GERD, bronchial asthma, sinusitis classification, allergic rhinitis, eosinophil count, neutrophil count, lymphocyte count, and total sinus score were independent risk factors for recurrence after ESS in CRSwNP patients.

GERD is a term used for discomfort and extraesophageal symptoms caused by reflux (10). Patients often present with oesophageal and extraesophageal symptoms, such as cough, asthma, pharyngitis, and sinusitis (10, 11). The results of this study also indicate that GERD is an independent risk factor for recurrence after ESS for CRSwNP. Some scholars also believe that GERD and the stimulation of digestive enzymes can cause damage to the upper airway mucosa; moreover, GERD may infiltrate through the nasopharynx, leading to mucosal infections in the nasopharynx (12, 13). This leads to recurrence after ESS for patients with CRSwNP (12–14). Patients with concomitant bronchial asthma exhibit the same inflammatory cytokine infiltration response in both the nasal sinus mucosa and bronchial mucosa. After simple surgical removal of nasal mucosal lesions, the lesions or inflammatory factors associated with bronchial asthma migrate to the sinuses, thereby inducing the recurrence of CRSwNP (15, 16). Lemmetyinen et al. (17) also reported that common comorbidities of asthma include acute sinusitis, CRSwNP, and GERD. This study revealed that concurrent asthma increases the risk of postoperative recurrence. Immunity, surgical time, and infection are closely related to sinus classification. For patients with Grades II and III sinusitis, due to the severity of the disease, more steroid drugs are needed for treatment, leading to a decrease in immune tolerance and increased susceptibility to pathogens, which is the main cause of disease recurrence (18, 19). Moreover, the surgery time for Grades II and III sinusitis is relatively long, causing damage to the body and increasing inflammatory factors, which can easily induce infection and cough, exacerbating the recurrence of the condition (20, 21). Allergic rhinitis is characterized mainly by symptoms such as sneezing, nasal itching, and nasal congestion, which are caused mainly by immunoglobulins (22, 23). Pressure on the nasal mucosa can cause continuous swelling, increased secretion of mucus, and congestion in the nasal cavity (23). Zhang et al. (24) reported that CRSwNP is closely related to allergic sinusitis and is the main risk factor. The results of this study also indicate that patients with concomitant allergic sinusitis have an 8.076-fold increase in the recurrence rate after ESS (OR: 9.076; 95% CI: 3.267–43.373). The pathological manifestation of CRSwNP nasal polyps is eosinophil-type nasal polyps, and there is a high probability of recurrence after surgery (25). Zhu et al. (26) reported that patients with eosinophil-type CRSwNP had higher risks of postoperative recurrence and treatment failure. Yang et al. (27) also reported that an increase in tissue eosinophils increases the probability of postoperative recurrence of ESS and can serve as an important predictive indicator for nasal polyp recurrence. Neutrophils can synthesize and release many cytokines and reactive oxygen species, which accumulate in the lesion tissue, causing significant thickening of the tissue basement membrane and an increase in the number of goblet cells, leading to severe damage to the epithelial protective barrier system (28, 29). Various pathogenic bacteria are more likely to colonize damaged epithelial cells, leading to repeated inflammatory infections. Nasal mucosal tissue can be stimulated directly or indirectly to produce a significant amount of active substances (29, 30). Ultimately, this leads to a significantly increased risk of recurrence after ESS in CRSwNP patients. In addition, the relative decrease in the peripheral blood lymphocyte count leads to a significant decrease in the patient’s cellular immune level. This ultimately leads to a significant decrease in the patient’s ability to resist inflammation and infection (28–30). Brescia et al. (31) also reported that the preoperative neutrophil-to-lymphocyte ratio was significantly greater in patients who experienced disease recurrence than in patients who did not. The results of this study also revealed that an increase in the neutrophil count and a decrease in the peripheral blood lymphocyte count are independent risk factors for recurrence after ESS for CRSwNP. In clinical practice, examination evidence from nasal endoscopy or sinus CT has become necessary for the diagnosis of CRSwNP. According to the Lund Mackay grading system, scoring sinus CT scans is a commonly used method for objective evaluation of sinusitis (32, 33). The results of this study indicate that patients with higher total sinus scores should maximize the preservation of normal tissue, which is beneficial for the repair of the mucosa and nasal cavity and reduces postoperative recurrence.

The nomogram model consists of three main parts: variable names, scale segments, and assigned values. Each variable has a corresponding dividing line segment. Each classification of variables has a corresponding score, which is concise, intuitive, and easy to understand and apply (34). Nomograms do not require complex calculations from traditional mathematical models and can quickly determine the risk of recurrence after ESS for CRSwNP through the use of auxiliary lines and simple summation calculations (34, 35). Medical staff can predict the incidence of recurrence after ESS for CRSwNP on the basis of the scores of various items of CRSwNP patients and identify high-risk patients with postoperative recurrence of CRSwNP as early as possible. Moreover, a certain degree of intervention should be taken for controllable risk factors to minimize the possibility of postoperative recurrence of CRSwNP. Du et al. (36) has developed a nomogram model based on blood eosinophils, total serum IgE level, asthma comorbidity, and the number of previous endoscopic sinus surgery, and the model showed good performance in preoperatively predicting CRSwNP Recurrence. In this study, we constructed a nomogram model based on the eight identified risk factors and multiple verifications were conducted to avoid overfitting of the model and ensure its accuracy. The results revealed that the C-index was 0.935 (95% CI: 0.902–0.968), the AUC was 0.948 (95% CI: 0.918–0.978), the sensitivity was 68.42%, the specificity was 95.85%, and the correction curve roughly matched the trend of the ideal curve. These findings indicate that the model constructed in this study has good predictive power for the risk of recurrence after ESS for CRSwNP patients.

However, this study has certain limitations: (1) the sample size included in this study is relatively small, and further expansion of the sample size is needed in the future; (2) this study did not include postoperative related biochemical indicators in the analysis, which may lead to bias in the research results. (3) While bronchial asthma was considered, aspirin intolerance was not included in the analysis. (4) This study was a retrospective analysis. The surgical procedures were not performed by the same team, which may have a certain impact on postoperative recurrence due to limitations in physician operating standards and other objective conditions. In addition, adherence to postoperative treatment may varied significantly, which may also affect postoperative recurrence. (5) This was a single-centre study involving internal validation on patients in tertiary hospitals. Therefore, it is currently unclear whether this model can be applied to external patients, especially those from secondary and primary health care institutions. To address these limitations, we should conduct large-scale prospective studies and external validation in secondary and primary health care institutions in future surveys.

## Conclusion

GERD, bronchial asthma, classification of sinusitis, allergic rhinitis, eosinophils, neutrophils, lymphocyte count, and total sinus score are independent risk factors for recurrence after ESS in CRSwNP patients. The prediction model performs well for identifying patients with blood CRSwNP at high risk for recurrence after ESS.

## Data Availability

The original contributions presented in the study are included in the article/Supplementary Material, further inquiries can be directed to the corresponding author.
